# Editorial: Highlights in Pain Mechanisms 2021/22

**DOI:** 10.3389/fpain.2023.1212137

**Published:** 2023-06-06

**Authors:** Aurélie Flaive, Alexia Coulombe-Leveque, Guillaume Leonard

**Affiliations:** ^1^ Research Centre on Aging, CIUSSS de l’Estrie-CHUS, Sherbrooke, QC, Canada; ^2^ Faculty of Medicine and Health Sciences, Université de Sherbrooke, Sherbrooke, QC, Canada; ^3^ School of Rehabilitation, Faculty of Medicine and Health Sciences, Université de Sherbrooke, Sherbrooke, QC, Canada

**Keywords:** neuroimmune response, chronic pain, chemokine, microvascular, parvalbumin, trigeminal pain, COVID-19, exercise

**Editorial on the Research Topic** Highlights in pain mechanisms 2021/22

## Introduction

Chronic pain is one of the most widespread health problems worldwide and its management remains suboptimal. Improving chronic pain management requires (i) a better understanding of the pathophysiological mechanisms underlying pain and (ii) the development of patient-specific treatments, based on the pathophysiological mechanisms involved. This article reviews 8 recently published papers that have made recent advances in identifying potential therapeutic targets and/or in applying new treatments based on these targets.

## Pathophysiological mechanisms underlying pain

The first topic of interest is parvalbumin-positive neurons, which could be associated with increased sensitivity to nociceptive stimuli (Miyahara et al.). In animal models, reserpine injections mimic fibromyalgia and increase parvalbumin-positive neuron density. Furthermore, in these models, optogenetic activation of parvalbumin neurons increases pain sensitivity to nociceptive stimuli. While parvalbumin neurons cannot be directly studied in humans, the observations of Miyahara and colleagues suggest that these neurons also drive the elevated gamma oscillations (which as noted by the authors are elevated and correlated to pain levels in fibromyalgia patients). These gamma oscillations could therefore serve as a biomarker for nociception, with the underlying parvalbumin-positive neurons offering a possible therapeutic target.

Also of interest are chemokines, a family of proteins involved in pain modulation in the peripheral and central nervous system (Solis-Castro et al.). In their review, Solis-Castro and colleagues summarize the current evidence for chemokine involvement in chronic orofacial (trigeminal) pain, going over 30 reports from animal research and 15 reports from human research. While current human research is only at the stage of chemokine polymorphism identification, many animal trials have tested chemokine antagonists with promising preliminary results. Their review provides a clear and detailed picture of the knowledge to date, as well as testable hypotheses to guide future research.

Continuing with chemokine involvement in chronic pain, Silva and Malcangio focus on a specific chemokine receptor—CX3CR1, which binds the CX3C chemokine known as fractalkine—and its role in microglia pro-inflammatory signaling in response to pain. This fractalkine/CX3CR1 pathway in the microglia is potentiated during the pro-inflammatory response observed following pain, resulting in enhanced communication between neurons and microglia; Silva and Malcangio theorize that this increase in communication underlies neuronal sensitization in neuropathic pain. Disruption or downregulation of this pathway could offer a possible treatment avenue for neuropathic pain.

In the peripheral nervous system, neuropathic pain arises as a result of sustained increased excitability and spontaneous neuronal activity in affected neurons (Alles and Smith). This abnormal activity is partly attributable to changes in Na+/Ca2+/K + ion channels. In their colossal review, Alles and Smith provide a detailed and comprehensive account of over 30 of these various channel subtypes, going over their specific roles as well as their pharmacological manipulation. They highlight that while pharmacological interventions targeting these channels seem promising for pain management, they have had a disappointing performance in the clinic. As such, Alles and Smith offer an insightful and thorough discussion of possible ways to optimize this treatment avenue, e.g., exploring the use of CRISPR technologies and lending more importance to sex-differences in pain etiology.

Moving away from the nervous system entirely, Coderre summarizes the current evidence on the microvascular system dysfunctions (arterioles, capillaries, and venules) that have been observed in patients with chronic pain, as well as the possible mechanisms by which these dysfunctions could lead to nociception. He cites for example the decreased nutritive blood flow in the skin and muscles of patients with complex regional pain syndrome and fibromyalgia that could contribute to oxidative stress and tissue ischemia. This review makes a compelling argument for future research on this topic, in both the fundamental and the clinical fields.

## Clinical case and understandings of pain

The recent COVID-19 pandemic has—unfortunately—opened up a whole new research avenue in pain etiology and treatment. Long-COVID specifically is often characterized by pain, among other neurological symptoms. In their case-report, Özkan et al. provide a fascinating account of a patient with severe and persistent migraine following COVID infection. After unsuccessful attempts to treat her migraines with usual care, Özkan et al. attempted a treatment using calcitonin gene-related peptide (CGRP) monoclonal antibodies, and obtained an immediate successful response, with the patient making a full recovery after 4 monthly doses. Based on previous research, the authors hypothesize that the mechanism underlying the patient's headaches (and the success of the treatment) is related to the similarities between CGRP receptor and SARS-CoV-2 spike proteins, leading to a sustained autoimmune response against CGRP-receptors.

Also related to COVID research is Savarraj et al.'s prospective study on pain 3 months after hospitalization for COVID. Their study was conducted on 58 patients—72% Latino, 53% with comorbid obesity, 38% with diabetes, and 46% with a history of hypertension—and found that 60% of them reported pain 3 months after hospitalization. Pain was not associated with severity of acute symptoms, although younger patients and women tended to be at higher risk of developing post-COVID pain. This report was one of the first looking at neurological symptoms in long-COVID.

In the field of rehabilitation, Mailloux et al. have demonstrated that sustained (4 min) isometric contractions of the wrist flexor muscles can induce widespread hypoalgesia. This effect was not replicated with sustained isometric contractions of low-back extensor muscles—perhaps because this muscle group, unlike wrist flexors, is quite used to (and physiologically adapted to) such sustained isometric contractions. While it remains uncertain whether more functional back exercises could induce a widespread hypoalgesia, Mailloux's study brings us one step closer to understanding exercise-induced hypoalgesia.

## Conclusion

While much remains to be discovered relating to pain, the 8 papers showcased in this editorial have made significant contributions to the field. By summarizing and advancing our knowledge of the neurological mechanisms underlying pain—from the involvement of parvalbumin-positive neurons, chemokines, ion channels, the microvascular system and CGRP receptors mimicking SARS-CoV-2 spike proteins to exercise-induced hypoalgesia—they pave the way for potential new treatment avenues for pain, bringing us one step closer to easing this global burden.

